# The Association of Transporter Genes Polymorphisms and Lung Cancer Chemotherapy Response

**DOI:** 10.1371/journal.pone.0091967

**Published:** 2014-03-18

**Authors:** Ying Wang, Ji-Ye Yin, Xiang-Ping Li, Juan Chen, Chen-Yue Qian, Yi Zheng, Yi-Lan Fu, Zi-Yu Chen, Hong-Hao Zhou, Zhao-Qian Liu

**Affiliations:** 1 Institute of Clinical Pharmacology, Hunan Key Laboratory of Pharmacogenetics, Central South University, Changsha, Hunan, P. R. China; 2 The Affiliated Cancer Hospital of XiangYa School of Medicine, Central South University, Changsha, Hunan, P. R. China; National Cancer Center, Japan

## Abstract

Lung cancer is one of the most common cancers and is the leading cause of death worldwide. Platinum-based chemotherapy is the main treatment method in lung cancer patients. Our previous studies indicated that single nucleotide polymorphisms (SNPs) in some transporter genes played important role in platinum-based chemotherapy efficacy. The aim of this study was to investigate the association of SNPs in transporter genes and platinum-based chemotherapy efficacy. The main polymorphisms on transporters *OCT2, LRP, AQP2, AQP9* and *TMEM205* genes were genotyped in 338 lung cancer patients. The rs195854 in genotypic model, rs896412 in genotypic and recessive models for all subjects showed significant association with chemotherapy response. In stratification analysis, *TMEM205* rs896412, *OCT2* rs1869641 and rs195854, *AQP9* rs1516400 and *AQP2* rs7314734 showed significant relation to chemotherapy response. In conclusion, the genetic polymorphisms in *OCT2*, A*QP2*, *AQP9* and *TMEM205* may contribute to chemotherapy response in lung cancer patients.

## Introduction

Lung cancer is one of the most common cancers and is the leading cause of death worldwide [1]. The percentage of five-years survival was about 15 which is much lower than other cancers [2]. Lung cancer consists of two types: non-small cell lung cancer (NSCLC) and small cell lung cancer (SCLC). Surgery is the mainstay of treatment for early NSCLC patients, while the later stage NSCLC and SCLC patients were mainly treated by platinum-based chemotherapy [3], [4]. Cisplatin, carboplatin were the most commonly used first-line clinical therapeutic drugs, but the tumors were always resistant to them and decreased their efficacy [4].

The chemotherapy resistance has several mechanisms, including reduced platinum compounds accumulation (decrease intake or increase efflux by transporters), detoxification, or increased level of DNA damage repair and so on [3], [5]. Several transporters contribute to platinum accumulation in the cancer cells. Aquaporins(AQPs)are members of a family of transmembrane proteins that have the function of transporting molecular water channels. The AQPs family contains at least 11 different types [6]. The expression of *AQP2* and *AQP9* were reduced in Pt resistant lines presenting that they are the potential new Pt drug transporters [7]. *TMEM205* was a novel transmembrane protein, having four transmembrane domains, is predicted to be a secretion-related protein by its nucleotide sequences [5]. The resistance to cisplatin was increased by transfecting *TMEM205* gene in cisplatin-resistant cells [8], [9]. Transmembrane protein 205 (*TMEM205*) may decrease the accumulation of platinum compound by increasing efflux [5]. The organic cation transporter 2 (*OCT2*), encoded by *SLC22A2* gene, had a potential role of increasing platinum uptake and sensitivity [10]–[12]. Resistance-related protein (*MVP/LRP*) can up-regulate sensitivity by increasing cellular cisplatin accumulation and/or by decreasing cisplatin efflux from nuclei in ovarian cancer cells [13]. To sum up, increasing the expression of *AQP2*, *AQP9*, *OCT2* and *MVP/LRP* or decreasing the expression of *TMEM205* might lead to platinum sensitivity.

Using the same drug and dose patients will have different response to chemotherapy because of comparable internal or external differences such as patient age, smoking status, their overall health and genetic variants [14]. Many studies found that SNPs in drug transporters play important role in metabolism and transport of therapeutic drugs, and affecting the response to therapy [15]. Daniele Campa et.al [16] investigated the relationship of *ABCB1, ABCC2* and *ABCG2* polymorphisms with platinum-based chemotherapy efficacy and demonstrated that a SNP of *ABCC2* was significantly linked with chemotherapy response. Our previous studies showed that several copper transporter protein1 SNPs may be significantly associated with platinum-based chemotherapy efficacy and toxicity in lung cancer patients [17], [18]. Oliver Zolk et.al indicated that the c.808 G>T SNP in *OCT2* significantly altered uptake of endogenous compounds and drugs [19]. Therefore we hypothesized that some other transporter gene polymorphisms may also be related with platinum-based chemotherapy efficacy.

In this work, we used the tagging method to analysis the relationship of 26 polymorphisms of *AQP2, AQP9, LRP/MVP, TMEM205* and *OCT2* genes with platinum-based chemotherapy response (Table 1). This the first study to investigate association of these genes polymorphisms with lung cancer chemotherapy response.

**Table 1:** The 26 gene polymorphisms examined in this study.

Gene	Location	dbSNP	Category	Call Rate (%)	Polymorphism	MAF
*LRP*	16q13.3	rs7204252	3′ downstream	98.52	C/T	0.081
		rs4788186	Intron	99.11	A/G	0.27
		rs4788184	nearGene-5	100.00	C/T	0.00
		rs1057451	Intron	99.70	G/T	0.088
*OCT2*	6q25.3	rs195862	5′ downstream	99.11	A/C	0.036
		rs195854	5′ downstream	90.23	A/T	0.27
		rs3823036	5′ downstream	99.11	C/T	0.30
		rs2444933	5′ downstream	98.82	C/T	0.27
		rs1883306	5′ downstream	93.20	A/C	0.32
		rs1869641	5′ downstream	98.52	C/T	0.17
*TMEM205*	19p13.2	rs7251786	3′ downstream	99.41	C/T	0.25
		rs896412	5′ downstream	99.41	C/G	0.19
		rs172731	5′ downstream	98.22	C/T	0.22
*AQP2*	12p13.2	rs461872	Intron	99.70	A/G	0.23
		rs3759125	nearGene-5	99.70	A/C	0.40
		rs7305534	nearGene-5	98.82	C/T	0.46
		rs296766	UTR-3	100.00	C/T	0.14
		rs3759126	nearGene-5	99.11	A/G	0.38
		rs7314734	nearGene-5	100.00	C/T	0.095
		rs10875989	UTR-3	98.82	C/T	0.40
*AQP9*	15q21.3	rs1867380	Exon	99.11	A/G	0.18
		rs1516400	nearGene-5	98.22	A/G	0.47
		rs1554203	nearGene-5	99.70	A/G	0.16
		rs9920375	3′ downstream	99.11	C/T	0.42
		rs2077737	nearGene-3	99.70	C/T	0.34
		rs8023369	3′ downstream	98.52	G/T	0.39

## Materials and Methods

### Study subjects

The study protocol has been approved by the Ethics Committee of Xiangya School of Medicine, Central South University with a registration number of CTXY-110008-2. The clinical research admission was approved by Chinese Clinical Trial Registry and the registration number is ChiCTR-RO-12002873 (http://www.chictr.org/usercenter/project/edit.aspx?proj=4039). Lung cancer patients were quantified and enrolled between September 2011 and March 2013 at Xiangya Hospital of Central South University and Hunan province tumor Hospital in Changsha Hunan. All the patients were provided written informed consent in compliance with the code of ethics of the World Medical Association (Declaration of Helsinki) before this study was initiated. The basic clinical characteristics were collected including age, smoking status, histology, gender and TNM stage, Eastern Cooperative Oncology Group Performance Status (ECOG PS).

The inclusion criteria were listed as followed: (1) Patients who were diagnosed lung cancer by histology or cytology; (2) Patients who had never received any radical or biological therapy before and during chemotherapy; (3) All patients who were received at least two cycle of first line chemotherapy; (4) All patients who were received throughout follow up among six months. The exclusion criteria were listed as followed: (1) Patients who were in pregnancy or feeding period; (2) Patients who had been diagnosed with other malignancies; (3) Patients who had brain metastases; (4) Patients who had active infection.

The enrolled patients in this study received first-line platinum-based chemotherapy including cisplatin and carboplatin. Chemotherapy response was assessed after first two cycles of chemotherapy according to the RECIST guideline (version 1.1) for solid tumors [20]. The patients that showed complete response (CR) or partial response (PR) were regarded as platinum sensitivity, while progressive disease (PD) or stable disease (SD) were regarded as platinum resistance [20].

### Tagging SNPs selection

We investigated all of the common genetic variants in *TMEM205, AQP2, AQP9, LRP* and *OCT2*. All SNPs of the 5 kb upstream of the first exon and 5 kb downstream of the last exon of the five genes were selected as the candidate SNPs. In total, 26 SNPs were selected in this work (Table 1). All these 26 SNPs satisfied the following criteria: (1) The SNPs were chosen from the International HapMap Project Phase II database of Chinese population (http://www.hapmap.org/); (2) All SNPs were haplotype tagger SNPs; (3) Minor allele frequency (MAF) was larger than 5% in Beijing Han population China; (4) The pairwise linkage disequilibrium was squared correlation coefficient (r^2^)>0.8. This work was performed by using Haploview version 4.2 [21].

There were 3 tagger SNPs for *TMEM205*, mean r2 of 0.988, 4 tagger SNPs for *LRP*, mean r2 of 1.000, 7 tagger SNPs for *AQP2*, mean r2 of 0.997, 6 tagger SNPs for *AQP9*, mean r2 of 0.964, 6 tagger SNPs for *OCT2*, mean r2 of 1.000.

### DNA extraction and genotyping procedure

Approximately 5 ml venous blood was collected from each patient for genetic studies. Genomic DNA was extracted from whole peripheral blood using either the DNeasy Blood & Tissue Kit (Qiagen, Shanghai China) or Genomic DNA Purification Kit (Promega, USA) according to the standard protocols.

Poymorphisms were detected by using Sequenom Mass Array Genotype Platform (Sequenom, San Diego, California, USA). Primers were designed by using AssayDesigner 3.1 software. Primer sequences were shown in Table S1. Procedures of genotyping are the following five main steps: (1) Polymerase chain reaction (PCR) amplification; Components and reagents were as following: 1.8 μL HPLC grade Water, 0.5 μL 10 × PCR Buffer with 15 mM MgCl2, 0.4 μL 25 mM MgCl2, 0.1 μL 25 mM dNTP Mix, 0.2 μL 0.5 mM Primer Mix, 0.2 μL 5 U/μL HotStar Taq and 1 μL 10 ng/μL DNA in an final volume 5 μL. PCR was ran at 94°C for 15 minutes, thermo cycling 45 cycles (94°C for 20 seconds, 56°C for 30 seconds and 72°C for 1 minutes), extension at 72°C for 3 min. (2) SAP treatment; Components and reagents were as following: 1.53 μL Nanopure Water, 0.17 μL SAP Buffer, 0.3 μL SAP Enzyme(1.7 U/μL) in an final volume 2 μL. The conditions were 37°C for 40 minutes, 85°C for 5 minutes. (3) Extension reaction;Components and reagents were as following: 0.619 μL Nanopure water, 0.2 μL iPLEX Buffer Plus, 0.2 μL iPLEX Termination mix, 0.94 μL iPLEX Extend Primer Mix, 0.041 μL iPLEX Enzyme, 2 μL SAP reagent and 5 μL PCR reagent in an final volume 9 μL. The conditions were 94°C for 30 seconds, 94°C for 5 seconds, 40 cycles of 52°C for 5 seconds, 5 cycles of 80°C for 5 seconds, and 72°C for 3 minutes. (4) Ion Exchange Clean-up; (5) Fragment analysis; Polymorphisms calls were analyzed by using Sequenom's Spectro Typer 4.0 software.

### Statistical analysis

All SNPs obeyed Hardy - Weinberg Equilibrium (HWE) by using the chi - square test. The association of genotypes and chemotherapy response were tested with logistic regression analysis.The potential covariates on chemotherapy response were selected by using binary logistic regression. Age, Sex, smoking status, PS and histology type were considered as covariates. All the above analysis were performed by plink (version 1.07, http://pngu.mgh.harvard.edu/purcell/plink/) and/or SPSS 13.0 (SPSS Inc, Chicago, Illinois, USA). Odds ratios (OR) and 95% confidence intervals (CI) were used to assess response to chemotherapy. P<0.05 was considered as significant. The figure was performed using StataSE version 12 (StataCorp, College Station, TX, USA). All data can be provided to anyone who wants via email.

## Results

A total of 338 patients had been studied. The basic clinical characteristics of these patients were shown in Table 2. The responders(CR+PR)were 154 and non-responders were 184 with the median age of 55.6±8.9 years (range 36–77 years) and 55.3±8.8 years (range 31–76 years), respectively. There were no significant differences between responders and non-responders in sex, age and smoking status indicated that the populations were adequately matched. The non-small cell lung carcinoma patients were more likely to be non-responders than responders (79.9 vs 57.8%, P<0.0001). The ratio of ECOG 0–1 was lower in responders than non-responders (96.8 vs 99.5%, P = 0.047).

**Table 2:** Main clinical characteristics of lung cancer patients.

Characteristics	Responders		Non-responders	
	N	(%)	N	(%)
Total	154	100.0	184	100.0
Age(years)				
≤55	72	53.2%	84	45.7%
>55	82	46.8%	100	54.3%
Gender				
Male	131	85.1%	140	76.1%
Female	23	14.9%	44	23.9%
Histology				
NSCLC	89	57.8%	147	79.9%
SCLC	65	42.2%	37	20.1%
Stage (non-small cell lung carcinoma)				
I–II	3	2.0%	6	3.3%
III–IV	86	55.8%	141	76.6%
Stage (small cell lung carcinoma)				
Limited	33	21.4%	14	7.6%
Extensive	32	20.8%	23	12.5%
Smoking				
Yes	105	68.2%	106	57.6%
No	49	31.8%	78	42.4%
ECOG PS				
0–1	149	96.8%	183	99.5%
>1	5	3.2%	1	0.5%
Chemotherapeutic regiment				
Cisplatin	119	77.3%	152	82.6%
Carboplatin	35	22.7%	32	17.4%

Covariates analysis showed that histology and ECOG might be statistically significant risk factors on platinum-based chemotherapy efficacy in all population, female and NSCLC patients. Histology was a statistically significant factor for male, no-smoking and smoking patients. Histology, gender and smoking or not might be statistically significant factors for patents above 55 years. All possible covariates were adjusted before data analysis. All 26 polymorphisms that probably were associated with platinum-based chemotherapy efficacy were detected and analyzed. The basic information of the 26 SNPs were shown in Table 1. However, to make the table not too complicate, only the most significant 5 SNPs' data were listed in the published version. The others results were listed in Table S2. We analyzed the association in genotypic, dominant and recessive models, respectively. The rs195854 (OR = 0.60, 95% CI, 0.36–1.00, P = 0.049) of *OCT2* might be associated with chemotherapy efficacy in genotypic model, rs896412 of *TMEM205* presented significance in genotypic (OR = 0.29, 95% CI, 0.10–0.81, P = 0.019) and recessive (OR = 0.082, 95% CI, 0.010–0.66, P = 0.019) models. There may be no polymorphisms significantly associated with chemotherapy efficacy in dominant model.

**Figure 1:**
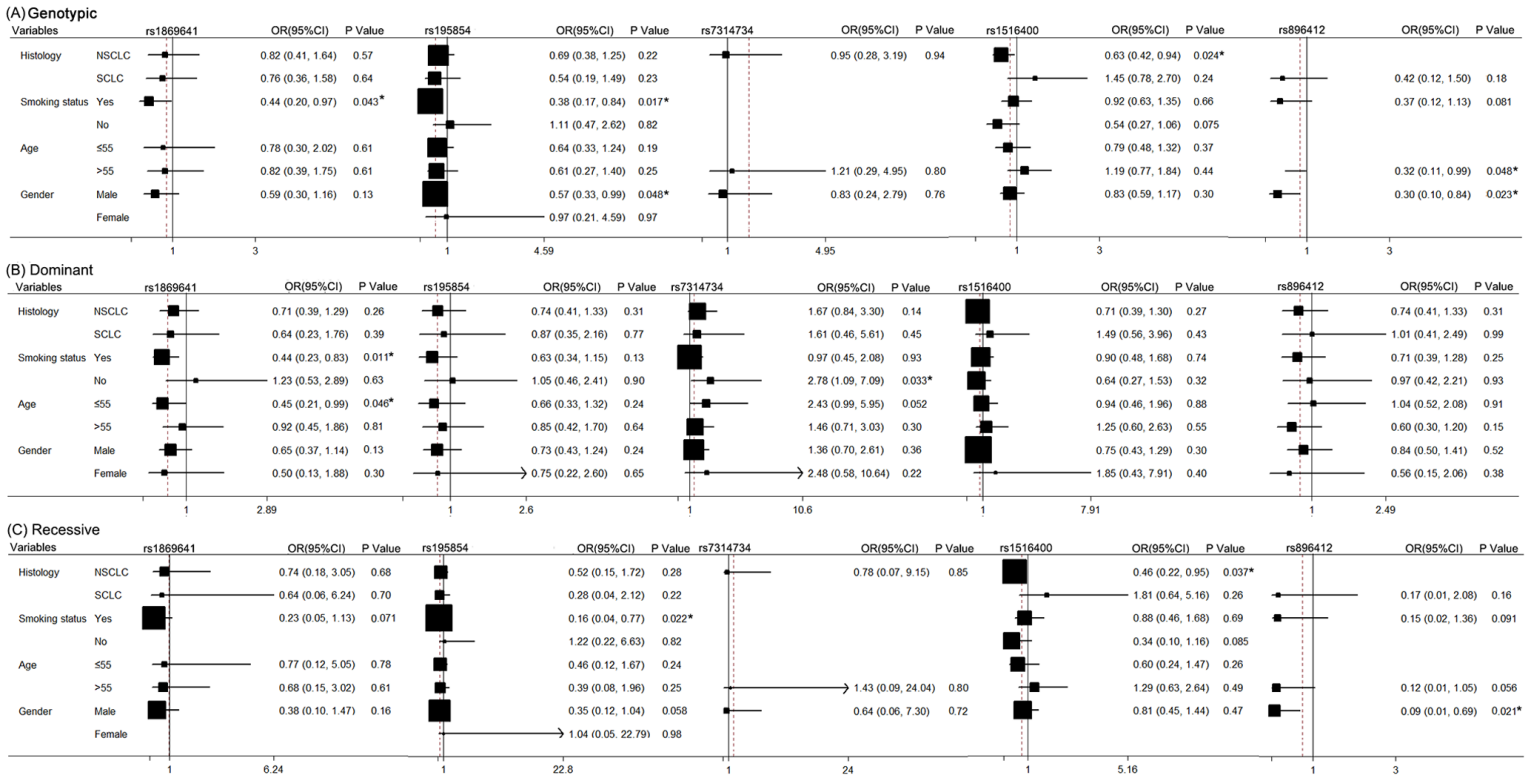
Stratification analysis of the associations of 5 SNPs and chemotherapy efficacy in Genotypic (A), Dominant (B), Recessive (C) models. Each box and horizontal line represents the OR and 95% CI. NSCLC: non-small cell lung carcinoma, SCLC: small cell lung carcinoma. *P<0.05.

Non-small cell and small cell lung cancer were very different biologically. Smokers and non-smokers, younger and elders, male and female also may be different in chemotherapy response. To further investigate the relationship of these SNPs with response of chemotherapy in these different categories, we performed the stratification analysis. Results of the five SNPs were shown in Figure 1. The others were listed in Table S3. The results showed that *OCT2* rs1869641 in genotypic and dominant models for smoke subjects and in dominant model for age ≤ 55 populations was likely significantly associated with chemotherapy efficacy. *OCT2* rs195854 in genotypic model for smoking and male subgroup, as well as in recessive model for smoking subgroup might be associated with chemotherapy response. The rs7314734 of *AQP2* only showed significant association with chemotherapy response for non-smokers in dominant model. The rs1516400 of *AQP9* might be significantly associated with chemotherapy for NSCLC patients in genotypic and recessive models. The rs896412 of *TMEM205* showed significant association for subgroup of age >55 in genotypic model, male subgroup in genotypic and recessive models.

In this study, the number of independent variables for *OCT2, LRP, AQP2, AQP9* and *TMEM205* are 6, 4, 7, 6 and 3, respectively. Each SNP was analyzed in three genetic models. To take into account the problem of multiple testing, the significance threshold is therefore 0.05/3(6+4+7+6+3) = 0.0006. Using this threshold, no one association remained significant.

## Discussion

In this study, we investigate the association of 5 transporter genes (*OCT2, AQP2, AQP9, MVP* and *TMEM205*) SNPs with platinum-based chemotherapy response. Our results showed that SNPs rs195854 (*OCT2*) and rs186941 (*OCT2*), rs7314734 (*AQP2*), rs1516400 (*AQP9*), and rs896412 (*TMEM205*) might be related with chemotherapy response in lung cancer patients.

*OCT2* was mainly expressed on the basolateral membrane which was the main site of cisplatin-induced renal toxicity [7], [22], [23]. Studies suggested that stably transfected *OCT2* into cell lines, the intracellular accumulation of platinum and the formation of Pt-DNA adducts were significantly increased [24]. These studies suggested that *OCT2* might be a key transporter for cisplatin and regulator in renal elimination of cisplatin. Based on the present study, the SNPs rs195854 and rs186941 of *OCT2* are both showed association with chemotherapy in smoking subgroup and/or in male populations. This result suggested that the *OCT2* gene polymorphisms might play a critical role in the platinum-based chemotherapy efficacy. However, the significant association was only occurred in rs195854 in genotypic model in all patients. Genotypic model is comparing the wild-type homozygous, heterozygous and mutant homozygous subjects. However, the other two models were comparing two genotypes and the remaining genotype. We hypothesis the possible reason is that the difference may be bigger in genotypic model if the number of one genotype subjects is significant different in two groups. However, the real reason should be investigated in the future studies. In smoking subgroup, rs195854 and rs1869641 both showed more chemotherapy resistance in two of the three models (Figure 1). There is possibility that smoking is overriding the potential effect of genetic variation in *OCT2* in regulation of transport of platinum. Smoking may lead to poor chemotherapy response in many reasons. Such as, cigarette smoke may be related to alter the lung metabolism of many endogenous compounds, the activities of many biotransforming enzymes in lung tissues and affect the rates of metabolism for several drugs [25]. And those light or never-smoking lung cancer patients had a higher ratio of *EGFR* mutations that had been linked to *EGFR-TKI* therapy response [26], [27]. In future treatment of lung cancer, we might distinguish subjects according to their smoking habits and apply different chemotherapeutic agents.

The SNP rs195854 might be significantly associated with chemotherapy response for male patients in genotypic model and had the trend in recessive model although there was no significant association. This result was consistent with retarder drug elimination in women and that the expression of *OCT2* was significantly lower in females in rabbits, mice, and rats [28]–[30]. It is thus tempting to speculate sex-dependent role for *OCT2* in chemotherapy response contributes to the different elimination of cisplatin. A significant association was also occurred in subgroup of age ≤ 55 for rs1869641. Kelly K. Filip ski et.al suggested that rs316019 of *OCT2* might be not associated with their studied pharmacokinetic variables [24]. Our results showed that *OCT2* might be associated with platinum-based chemotherapy efficacy, but the specific mechanisms need to be studied in the further work.

*AQP9* was not only important to arsenic resistance in human lung cancer cells by enhancing arsenic uptake [31], but also play a critical role in development of platinum-based chemotherapy response. *AQP9* rs1516400 might be significantly associated with chemotherapy response for NSCLC patients in genotypic and recessive models but not for SCLC patients. Those results may be due to the NSCLC and SCLC differential various points, for example they had different molecular genetic abnormalities: the p53 mutation is higher in SCLC [32] and NSCLC are often over-expression in COX-2 [33]. Moreover, there are different in loss of cell cycle controls for the two types of histology [34], [35]. The different effect for *AQP9* rs1516400 in NSCLC and SCLC may reinforce the fact that they have different genetic prognostic markers. This studies identified that *AQP9* might be a potential predictive biomarkers for platinum-based chemotherapy response in NSCLC patients. However, the mechanism how *AQP9* play a role in platinum-based chemotherapy efficacy for lung cancer patients requires to be studied in the next work.

*AQP2* is the most important water channel in the apical plasma membrane [6]. It was found to be associated with resistance of cisplatin and might be a membrane transporter/binding protein/carrier for it [36]. The SNP rs7314734 of *AQP*2 showed more association with chemotherapy response in non-smoking subgroup and the reason could be explained similar to *OCT2*.

Ding-Wu Shen et.al showed that the expression of *TMEM205* was increased in cisplatin resistant cells [8]. However, once the gene was overexpressed cells got more resistant to cisplatin [9]. This study showed that *TMEM205* rs896412 might be significantly associated with chemotherapy response in all lung cancer patients (genotypic and recessive models) (Table 3), male subgroup (genotypic and recessive models) and subgroup of age >55 (genotypic model) (Figure 1). The different results in male and female subgroups may be linked with differ elimination of platinum similar to *OCT2*. Studies suggested that elder patients (>70 years) especially ones that had no coexistent diseases can get similar results from treatment as younger patients (<70 years) [37]–[39]. We found a statistically significant different between rs1869641 of *OCT2* in subgroup of age ≤ 55 and rs896412 of *TMEM205* in subgroup of age > 55. That was different comparing with the previous studies. The possible reason may be the different borderline (55, 70, respectively), but it should be further investigated. We thought that *TMEM205* may play an important role in lung cancer platinum-based chemotherapy response and would also be a possible biomarker for lung cancer chemotherapy.

**Table 3:** Association of 5-based chemotherapy response in all lung cancer patients.

Gene	Polymorphisms	Genotype	Responders	Non-responders	Genotypic		Dominant		Recessive	
			N(%)	N(%)	OR(95%CI)	P value	OR(95%CI)	P value	OR(95%CI)	P value
*OCT2*	rs1869641	GG	112(75.1)	120(65.22)	0.78(0.44–1.40)	0.41	0.67(0.40–1.10)	0.11	0.68(0.21–2.16)	0.51
		AG	32(21.48)	55(29.89)						
		AA	5(3.36)	9(4.89)						
	rs195854	AA	81(55.86)	81(50.63)	0.60(0.36–1.00)	0.049*	0.78(0.48–1.25)	0.30	0.38(0.14–1.02)	0.056
		AT	58(40.00)	63(39.38)						
		TT	6(4.14)	16(10.00)						
*AQP2*	rs7314734	CC	122(79.22)	155(84.24)	0.93(0.28–3.12)	0.90	1.49(0.84–2.67)	0.17	0.80(0.071–8.99)	0.86
		CT	31(20.13)	27(14.67)						
		TT	1(0.65)	2(1.09)						
*AQP9*	rs1516400	TT	46(30.67)	49(29.92)	0.80(0.58–1.10)	0.18	0.84(0.51–1.38)	0.49	0.66(0.38–1.15)	0.14
		CT	73(48.67)	86(47.25)						
		CC	31(20.67)	47(25.82)						
*TMEM205*	rs896412	GG	103(67.32)	115(62.84)	0.29(0.10–0.81)	0.019*	0.81(0.50–1.29)	0.37	0.082(0.010–0.66)	0.019*
		CG	49(32.03)	56(30.60)						
		CC	1(0.65)	12(6.56)						

*P<0.05.

The rs195854 and rs1869641 of *OCT2*, rs896412 of *TMEM205*, rs7314734 of *AQP*2, located in 5 kb upstream of the gene regions, the possible mechanism of regulating the expression were not known at present. We hypothesize that they locate in the enhancer area and the variant can regulate the expression. The rs1516400 located in the neargene-5 of *AQP9*. It may be in the promoter area and its mutation may regulate the activity of the promoter of *AQP9*, and then the expression of the *AQP9* was changed. However, the possible mechanisms need to be studied in the further studies.

This study has some limitations. On one hand, to get more credible conclusion, we should do an independent validation for these SNPs. We are thus trying to amplify the sample size and will validate these SNPs in the future study. However, the samples are very difficult to collect and not enough for another independent population validation now. On the other hand, evaluating the chemotherapy response after every two cycles of chemotherapy treatment is likely to get more reliable results. However, we can only unify the two cycles of chemotherapy for these patients. Many patients didn't receive more than four cycles of chemotherapy because of many reasons, such as financial condition and so on.

There was not any study indicated whether polymorphisms of these five genes were related to platinum-based chemotherapy response in lung cancer patients before. This study was the first one to investigate their associations with platinum-based chemotherapy response. The results of *TMEM205* rs896412, *OCT2* rs1869641 and rs195854, *AQP9* rs1516400 and *AQP2* rs7314734 suggested that these SNPs might be associated with platinum-based chemotherapy response. Further studies how these SNPs impact the platinum-based chemotherapy response for lung cancers should be warranted.

## Supporting Information

Table S1 **Primers of all the selected SNPs.**
(DOCX)

Table S2 **Association of the other single nucleotide polymorphisms and platinum-based chemotherapy response in all lung cancer patients.**
(DOCX)

Table S3 **Stratification analyses of the associations of the other polymorphisms and chemotherapy efficacy in genotypic, dominant, recessive models.**
(DOCX)
