# Supplementary material for: The Association of Transporter Genes Polymorphisms and Lung Cancer Chemotherapy Response
Source: PLoS One. 2014 Mar 18;9(3):e91967. doi: 10.1371/journal.pone.0091967 (PMC3958404; doi:10.1371/journal.pone.0091967)
Supplement: Table S1 — Primers of all the selected SNPs. (DOCX) [file pone.0091967.s001.docx]

Table S1. Primers of all the selected SNPs

| SNP ID | 2nd-PCRP | 1st-PCRP |
| --- | --- | --- |
| rs1057451 | ACGTTGGATGGCTCAGTCCTATGATTAGGC | ACGTTGGATGAGAACCGCCTAGATCAGAAG |
| rs10875989 | ACGTTGGATGACCTCTTCTTACCCTGAATG | ACGTTGGATGAAGGAGAGAATGGATAGGGC |
| rs1516400 | ACGTTGGATGGGTGTGGTCATAGGTAACTC | ACGTTGGATGTGCAGAACCAGGACAAAACC |
| rs1554203 | ACGTTGGATGTAATCAGAAACTAGGCCTGC | ACGTTGGATGCCTTGTTTTTCAAAACTGGC |
| rs172731 | ACGTTGGATGAAAAGAAAGGTCCCAGGTGC | ACGTTGGATGTCCCCTCTAAGATGGTACCC |
| rs1867380 | ACGTTGGATGTGAAATCCACCATCCAGAGC | ACGTTGGATGGCCACTACATGATGACACTG |
| rs1869641 | ACGTTGGATGCCAAGCATATGACGTAGGAG | ACGTTGGATGGCAAATTCCTAAATGTGCATC |
| rs1883306 | ACGTTGGATGTGAGCTCTCGCTGGTTTTAG | ACGTTGGATGAAAGAATGGGCCCCAGAGC |
| rs195854 | ACGTTGGATGCCTAGAATTCTGATTGTTGG | ACGTTGGATGGATGCACTTTTACTGGTGTA |
| rs195862 | ACGTTGGATGACAAGTGTCTCTTGCTCCAG | ACGTTGGATGAAAAGGAAGAGAGCCTGTGC |
| rs2077737 | ACGTTGGATGTGCTTTTGTTCCAGGTGCTC | ACGTTGGATGCTCCACAAAGGTAAATCCAC |
| rs2444933 | ACGTTGGATGTGGAGGTAGCAGCTGGTTTA | ACGTTGGATGAACATGATCCTCTAAGGTGG |
| rs296766 | ACGTTGGATGAGTCTGATGCTCAACATCCC | ACGTTGGATGCAGGACTCTCCTTTGCTTTG |
| rs3759125 | ACGTTGGATGTTTCTAGGACCTGTCACAGC | ACGTTGGATGTGCACTTTGAGGAGAACAGG |
| rs3759126 | ACGTTGGATGAAACAGCAGCTCCTAGAGCC | ACGTTGGATGGAATGGACACCCTCAGCTTC |
| rs3823036 | ACGTTGGATGTGCAGCTTCCTGATGCTTAG | ACGTTGGATGTTTTTCCTCTTTCCTGATGG |
| rs461872 | ACGTTGGATGCCAACAGAAACCTGGATGAG | ACGTTGGATGAAAGCAGGATGCAGACAGAC |
| rs4788184 | ACGTTGGATGTCTTGGCTCATTGCAACCTC | ACGTTGGATGCCTGTAATCCCAGCTACTTG |
| rs4788186 | ACGTTGGATGGCTAGGATTCGGCCATAAAC | ACGTTGGATGCAGCCCAGGATTACTAAAGC |
| rs7204252 | ACGTTGGATGCAACTAATCTGTGTCCCAGC | ACGTTGGATGTCCACTTCCATTCCATTGGC |
| rs7251786 | ACGTTGGATGTTGGGTAAAATGCAAGGGTC | ACGTTGGATGAAACCATGACCCCCAATTCC |
| rs7305534 | ACGTTGGATGTTCTGATCTGGCCCCTAGTC | ACGTTGGATGCCTGGCATGAAGGGAAAGTC |
| rs7314734 | ACGTTGGATGGGTGTATCTCGTTTCTCATC | ACGTTGGATGTTAGGTATGCGCAGGCAATG |
| rs8023369 | ACGTTGGATGTGTGCAACTGAGAAGGATTG | ACGTTGGATGGTGGGAGTGTGAAAACATGG |
| rs896412 | ACGTTGGATGGTTCAAAGTGCAGGAAAGGG | ACGTTGGATGGGCATTGACAACACATTGGC |
| rs9920375 | ACGTTGGATGTCATTGTCTTGCACCCTCAG | ACGTTGGATGGCATTTTCCTTTTTGCATC |
