# Supplementary material for: The Association of Transporter Genes Polymorphisms and Lung Cancer Chemotherapy Response
Source: PLoS One. 2014 Mar 18;9(3):e91967. doi: 10.1371/journal.pone.0091967 (PMC3958404; doi:10.1371/journal.pone.0091967)
Supplement: Table S2 — Association of the other single nucleotide polymorphisms and platinum-based chemotherapy response in all lung cancer patients. (DOCX) [file pone.0091967.s002.docx]

Table S2. Association of the other single nucleotide polymorphisms and platinum-based chemotherapy response in all lung cancer patients.

| Gene | Polymorphisms | Genotype | Responders | Non-responders | Genotypic | | Dominant | | Recessive | |
| --- | --- | --- | --- | --- | --- | --- | --- | --- | --- | --- |
|  |  |  |  | N(%) | OR(95%CI) | P value | OR(95%CI) | P value | OR(95%CI) | P value |
| OCT2 | rs3823036 | GG | 78 | 91 | 0.85(0.56-1.27) | 0.42 | 0.90(0.58-1.41) | 0.66 | 0.73(0.33-1.61) | 0.44 |
|  |  | AG | 64 | 74 |  |  |  |  |  |  |
|  |  | AA | 12 | 19 |  |  |  |  |  |  |
|  | rs2444933 | GG | 9 | 15 | 0.85(0.54-1.34) | 0.50 | 0.88(0.56-1.39) | 0.58 | 0.76(0.31-1.84) | 0.54 |
|  |  | AG | 58 | 73 |  |  |  |  |  |  |
|  |  | AA | 83 | 96 |  |  |  |  |  |  |
|  | rs1883306 | GG | 16 | 19 | 1.06(0.72-1.57) | 0.75 | 0.99(0.62-1.59) | 0.97 | 1.15(0.55-2.42) | 0.70 |
|  |  | GT | 57 | 77 |  |  |  |  |  |  |
|  |  | TT | 62 | 84 |  |  |  |  |  |  |
| LRP | rs7204252 | CC | 3 | 2 | 1.43(0.56-3.62) | 0.45 | 1.05(0.56-1.98) | 0.88 | 2.05(0.32-13.14) | 0.45 |
|  |  | CT | 20 | 24 |  |  |  |  |  |  |
|  |  | TT | 128 | 157 |  |  |  |  |  |  |
|  | rs4788186 | GG | 12 | 13 | 1.06(0.69-1.65) | 0.77 | 0.91(0.58-1.43) | 0.67 | 1.21(0.52-2.81) | 0.67 |
|  |  | AG | 55 | 76 |  |  |  |  |  |  |
|  |  | AA | 86 | 93 |  |  |  |  |  |  |
|  | rs1057451 | GG | 126 | 152 | NA | NA | 1.24(0.69-2.22) | 0.48 | NA | NA |
|  |  | GT | 27 | 32 |  |  |  |  |  |  |
|  |  | TT | 0 | 0 |  |  |  |  |  |  |
| AQP2 | rs1087598 | CC | 47 | 73 | 1.34(0.95-1.87) | 0.09 | 1.42(0.88-2.29) | 0.15 | 1.53(0.83-2.80) | 0.17 |
|  |  | CT | 73 | 86 |  |  |  |  |  |  |
|  |  | TT | 30 | 25 |  |  |  |  |  |  |
|  | rs3759125 | CC | 55 | 73 | 1.25(0.90-1.73) | 0.18 | 1.11(0.70-1.76) | 0.67 | 1.19(0.61-2.32) | 0.62 |
|  |  | CA | 67 | 84 |  |  |  |  |  |  |
|  |  | AA | 32 | 26 |  |  |  |  |  |  |
|  | rs461872 | GG | 90 | 108 | 1.25(0.76-2.06) | 0.38 | 0.96(0.61-1.52) | 0.87 | 1.64(0.61-4.37) | 0.33 |
|  |  | GA | 53 | 68 |  |  |  |  |  |  |
|  |  | AA | 10 | 8 |  |  |  |  |  |  |
|  | rs7305534 | TT | 41 | 60 | 1.12(0.82-1.54) | 0.47 | 1.32(0.80-2.17) | 0.17 | 1.05(0.61-1.79) | 0.86 |
|  |  | CT | 76 | 82 |  |  |  |  |  |  |
|  |  | CC | 36 | 39 |  |  |  |  |  |  |
|  | rs296766 | CC | 107 | 140 | 1.20(0.54-2.65) | 0.65 | 1.30(0.79-2.16) | 0.30 | 1.34(0.28-6.47) | 0.71 |
|  |  | CT | 43 | 41 |  |  |  |  |  |  |
|  |  | TT | 4 | 3 |  |  |  |  |  |  |
|  | rs3759126 | GG | 20 | 23 | 1.06(0.74-1.53) | 0.74 | 0.96(0.60-1.53) | 0.86 | 1.19(0.61-2.32) | 0.62 |
|  |  | GA | 74 | 97 |  |  |  |  |  |  |
|  |  | AA | 57 | 64 |  |  |  |  |  |  |
| AQP9 | rs2077737 | GG | 17 | 25 | 0.86(0.60-1.24) | 0.41 | 0.90(0.57-1.41) | 0.65 | 0.76(0.38-1.51) | 0.43 |
|  |  | GA | 68 | 78 |  |  |  |  |  |  |
|  |  | AA | 68 | 81 |  |  |  |  |  |  |
|  | rs9920375 | CC | 27 | 32 | 0.90(0.64-1.25) | 0.53 | 0.81(0.50-1.30) | 0.37 | 0.92(0.50-1.67) | 0.77 |
|  |  | CT | 71 | 92 |  |  |  |  |  |  |
|  |  | TT | 53 | 60 |  |  |  |  |  |  |
|  | rs1554203 | TT | 108 | 130 | 1.18(0.61-2.27) | 0.62 | 0.96(0.58-1.57) | 0.87 | 1.43(0.39-5.26) | 0.59 |
|  |  | CT | 40 | 49 |  |  |  |  |  |  |
|  |  | CC | 5 | 5 |  |  |  |  |  |  |
|  | rs1867380 | GG | 105 | 119 | 0.87(0.41-1.85) | 0.71 | 0.87(0.54-1.40) | 0.57 | 0.78(0.17-3.52) | 0.75 |
|  |  | GA | 44 | 59 |  |  |  |  |  |  |
|  |  | AA | 3 | 5 |  |  |  |  |  |  |
|  | rs8023369 | TT | 55 | 61 | 1.03(0.72-1.48) | 0.86 | 0.95(0.59-1.52) | 0.82 | 1.12(0.58-2.16) | 0.73 |
|  |  | GT | 74 | 98 |  |  |  |  |  |  |
|  |  | GG | 22 | 23 |  |  |  |  |  |  |
| TMEM205 | rs172731 | TT | 93 | 108 | 0.73(0.41-1.31) | 0.29 | 0.87(0.55-1.38) | 0.55 | 0.55(0.18-1.74) | 0.31 |
|  |  | TC | 53 | 63 |  |  |  |  |  |  |
|  |  | CC | 5 | 10 |  |  |  |  |  |  |
|  | rs7251786 | TT | 8 | 11 | 0.87(0.53-1.45) | 0.60 | 1.06(0.67-1.67) | 0.80 | 0.73(0.27-1.98) | 0.54 |
|  |  | CT | 60 | 67 |  |  |  |  |  |  |
|  |  | CC | 85 | 105 |  |  |  |  |  |  |
