# Supplementary material for: The Association of Transporter Genes Polymorphisms and Lung Cancer Chemotherapy Response
Source: PLoS One. 2014 Mar 18;9(3):e91967. doi: 10.1371/journal.pone.0091967 (PMC3958404; doi:10.1371/journal.pone.0091967)
Supplement: Table S3 — Stratification analyses of the associations of the other polymorphisms and chemotherapy efficacy in genotypic, dominant, recessive models. (DOCX) [file pone.0091967.s003.docx]

Table S3. Stratification analyses of the associations of the other polymorphisms and chemotherapy efficacy in genotypic, dominant, recessive models.

| Variables | Gene | Polymorphisms | Genotypic | | Dominant | | Recessive | |
| --- | --- | --- | --- | --- | --- | --- | --- | --- |
|  |  |  | OR(95%CI) | P value | OR(95%CI) | P value | OR(95%CI) | P value |
| NSCLC | OCT2 | rs3823036 | 0.95(0.59-1.53) | 0.84 | 0.89(0.52-1.53) | 0.67 | 1.00(0.39-2.54) | 0.99 |
|  |  | rs2444933 | 0.88(0.52-1.49) | 0.64 | 0.76(0.44-1.33) | 0.34 | 0.88(0.31-2.51) | 0.81 |
|  |  | rs1883306 | 1.06(0.68-1.66) | 0.78 | 0.96(0.54-1.71) | 0.89 | 1.12(0.47-2.67) | 0.81 |
|  | LRP | rs7204252 | 1.29(0.48-3.47) | 0.62 | 1.06(0.47-2.40) | 0.89 | 2.06(0.27-15.52) | 0.48 |
|  |  | rs4788186 | 1.00(0.61-1.63) | 0.99 | 0.73(0.42-1.26) | 0.26 | 1.33(0.51-3.53) | 0.56 |
|  |  | rs1057451 | NA | NA | 1.07(0.55-2.10) | 0.84 | NA | NA |
|  | AQP2 | rs1087598 | 1.26(0.84-1.88) | 0.27 | 1.44(0.80-2.59) | 0.23 | 1.07(0.50-2.25) | 0.87 |
|  |  | rs3759125 | 1.19(0.82-1.74) | 0.36 | 1.06(0.60-1.85) | 0.85 | 1.27(0.63-2.59) | 0.50 |
|  |  | rs461872 | 1.20(0.68-2.13) | 0.53 | 0.94(0.54-1.65) | 0.84 | 1.29(0.40-4.03) | 0.68 |
|  |  | rs7305534 | 1.12(0.77-1.64) | 0.56 | 1.21(0.66-2.22) | 0.54 | 1.09(0.56-2.13) | 0.79 |
|  |  | rs296766 | 1.30(0.48-3.51) | 0.60 | 1. 06(0.57-1.97) | 0.85 | 1.25(0.19-9.34) | 0.83 |
|  |  | rs3759126 | 1.11(0.73-1.69) | 0.62 | 1.17(0.65-2.09) | 0.60 | 1.30(0.59-2.87) | 0.52 |
|  | AQP9 | rs2077737 | 0.79(0.50-1.25) | 0.32 | 0.93(0.54-1.61) | 0.79 | 0.63(0.26-1.53) | 0.31 |
|  |  | rs9920375 | 0.84(0.56-1.27) | 0.42 | 0.79(0.45-1.40) | 0.42 | 0.75(0.34-1.65) | 0.48 |
|  |  | rs1554203 | 1.31(0.64-2.66) | 0.46 | 1.14(0.63-2.08) | 0.66 | 1.84(0.43-7.78) | 0.41 |
|  |  | rs1867380 | 0.54(0.18-1.60) | 0.27 | 0.71(0.40-1.28) | 0.26 | 0.32(0.035-2.85) | 0.31 |
|  |  | rs8023369 | 1.25(0.81-1.95) | 0.31 | 1.19(0.66-2.14) | 0.57 | 1.49(0.66-3.36) | 0.33 |
|  | TMEM205 | rs172731 | 0.80(0.40-1.61) | 0.54 | 0.80(0.45-1.42) | 0.44 | 0.69(0.17-2.86) | 0.61 |
|  |  | rs7251786 | 1.08(0.59-1.96) | 0.81 | 1.01(0.58-1.75) | 0.99 | 1.07(0.31-3.73) | 0.91 |
| SCLC | OCT2 | rs3823036 | 0.75(0.34-1.65) | 0.47 | 1.19(0.49-2.91) | 0.70 | 0.48(0.10-2.17) | 0.34 |
|  |  | rs2444933 | 0.94(0.37-2.44) | 0.91 | 1.54(0.62-3.84) | 0.35 | 0.71(0.11-4.46) | 0.71 |
|  |  | rs1883306 | 1.18(0.47-2.97) | 0.72 | 0.85(0.34-2.16) | 0.74 | 1.58(0.27-9.29) | 0.61 |
|  | LRP | rs7204252 | NA | NA | 1.35(0.43-4.28) | 0.61 | NA | NA |
|  |  | rs4788186 | 1.13(0.41-3.11) | 0.81 | 1.36(0.56-3.31) | 0.50 | 1.17(0.16-8.67) | 0.88 |
|  |  | rs1057451 | NA | NA | 1.57(0.36-6.80) | 0.54 | NA | NA |
|  | AQP2 | rs1087598 | 2.09(0.97-4.49) | 0.061 | 1.67(0.67-4.17) | 0.27 | 3.79(0.91-15.82) | 0.068 |
|  |  | rs3759125 | 2.07(0.96-4.45) | 0.063 | 1.60(0.64-3.96) | 0.31 | 3.86(0.92-16.25) | 0.78 |
|  |  | rs461872 | 1.89(0.52-6.83) | 0.33 | 0.94(0.37-2.36) | 0.90 | 3.89(0.31-49.14) | 0.29 |
|  |  | rs7305534 | 1.27(0.71-2.28) | 0.41 | 1.80(0.69-4.70) | 0.23 | 1.12(0.41-3.03) | 0.83 |
|  |  | rs296766 | 1.61(0.41-6.30) | 0.49 | 2.78(0.97-7.95) | 0.057 | 2.08(0.14-31.2) | 0.60 |
|  |  | rs3759126 | 0.75(0.36-1.54) | 0.43 | 0.51(0.20-1.31) | 0.16 | 0.83(0.22-3.09) | 0.78 |
|  | AQP9 | rs2077737 | 1.00(0.50-1.99) | 0.99 | 0.96(0.38-2.41) | 0.93 | 1.02(0.28-3.68) | 0.98 |
|  |  | rs9920375 | 1.06(0.56-1.99) | 0.87 | 0.91(0.34-2.40) | 0.85 | 1.25(0.42-3.74) | 0.69 |
|  |  | rs1554203 | 1.05(0.23-4.88) | 0.95 | 0.85(0.33-2.22) | 0.75 | 1.19(0.056-25.00) | 0.91 |
|  |  | rs1867380 | NA | NA | 2.14(0.78-5.89) | 0.14 | NA | NA |
|  |  | rs8023369 | 0.62(0.33-1.19) | 0.15 | 0.39(0.15-1.05) | 0.064 | 0.64(0.21-1.99) | 0.44 |
|  | TMEM205 | rs172731 | 0.72(0.26-1.97) | 0.52 | 1.35(0.563.28) | 0.51 | 0.43(0.058-3.11) | 0.40 |
|  |  | rs7251786 | 0.73(0.31-1.73) | 0.47 | 1.54(0.63-3.75) | 0.35 | 0.41(0.075-2.25) | 0.31 |
| <55 | OCT2 | rs3823036 | 0.89(0.51-1.56) | 0.69 | 0.90(0.47-1.73) | 0.75 | 0.82(0.28-2.42) | 0.73 |
|  |  | rs2444933 | 0.70(0.36-1.36) | 0.30 | 0.73(0.37-1.43) | 0.36 | 0.54(0.15-1.96) | 0.35 |
|  |  | rs1883306 | 1.16(0.67-2.03) | 0.60 | 1.37(0.69-2.75) | 0.37 | 1.14(0.40-3.23) | 0.81 |
|  | LRP | rs7204252 | 1.31(0.47-3.66) | 0.61 | 1.08(0.44-2.64) | 0.87 | 1.71(0.22-13.40) | 0.61 |
|  |  | rs4788186 | 0.58(0.29-1.18) | 0.13 | 0.77(0.40-1.50) | 0.45 | 0.35(0.088-1.41) | 0.14 |
|  |  | rs1057451 | NA | NA | 2.01(0.83-4.90) | 0.32 | NA | NA |
|  | AQP2 | rs1087598 | 1.27(0.77-2.10) | 0.36 | 1.43(0.71-2.86) | 0.32 | 1.32(0.53-3.27) | 0.54 |
|  |  | rs3759125 | 1.2(0.73-1.97) | 0.47 | 1.28(0.65-2.56) | 0.48 | 1.27(0.52-3.12) | 0.60 |
|  |  | rs461872 | 1.74(0.83-3.71) | 0.15 | 1.04(0.52-2.08) | 0.91 | 3.23(0.74-14.13) | 0.12 |
|  |  | rs7305534 | 1.24(0.78-1.97) | 0.37 | 1.18(0.57-2.43) | 0.66 | 1.50(0.67-3.32) | 0.32 |
|  |  | rs296766 | 1.01(0.36-2.82) | 0.99 | 1.26(0.60-2.65) | 0.54 | 0.95(0.13-7.32) | 0.96 |
|  |  | rs3759126 | 0.81(0.48-1.39) | 0.45 | 0.65(0.32-1.30) | 0.22 | 0.85(0.32-2.27) | 0.75 |
|  | AQP9 | rs2077737 | 0.93(0.52-1.66) | 0.80 | 0.84(0.43-1.63) | 0.60 | 0.95(0.31-2.86) | 0.92 |
|  |  | rs9920375 | 0.88(0.53-1.49) | 0.64 | 0.75(0.37-1.49) | 0.41 | 0.94(0.37-2.42) | 0.90 |
|  |  | rs1554203 | 0.55(0.17-1.77) | 0.32 | 0.81(0.40-1.66) | 0.56 | 0.32(0.032-3.22) | 0.33 |
|  |  | rs1867380 | 1.58(0.46-5.46) | 0.47 | 0.58(0.28-1.19) | 0.14 | 3.07(0.26-36.25) | 0.37 |
|  |  | rs8023369 | 0.97(0.59-1.58) | 0.90 | 0.89(0.45-1.76) | 0.74 | 1.01(0.41-2.48) | 0.99 |
|  | TMEM205 | rs172731 | 0.96(0.41-2.25) | 0.93 | 0.75(0.38-1.48) | 0.40 | 1.03(0.19-5.53) | 0.97 |
|  |  | rs7251786 | 1.41(0.67-2.96) | 0.36 | 0.65(0.33-1.28) | 0.21 | 2.42(0.56-10.47) | 0.24 |
| >55 | OCT2 | rs3823036 | 0.78(0.42-1.43) | 0.41 | 0.91(0.48-1.75) | 0.78 | 0.60(0.19-1.95) | 0.40 |
|  |  | rs2444933 | 1.03(0.54-1.96) | 0.92 | 1.00(0.52-1.92) | 0.99 | 1.08(0.31-3.72) | 0.91 |
|  |  | rs1883306 | 0.89(0.49-1.66) | 0.73 | 0.70(0.35-1.42) | 0.33 | 0.95(0.29-3.09) | 0.93 |
|  | LRP | rs7204252 | NA | NA | 0.97(0.38-2.48) | 0.95 | NA | NA |
|  |  | rs4788186 | 1.89(0.89-4.00) | 0.098 | 0.98(0.51-1.90) | 0.96 | 3.93(0.90-17.11) | 0.069 |
|  |  | rs1057451 | NA | NA | 0.74(0.31-1.76) | 0.50 | NA | NA |
|  | AQP2 | rs1087598 | 1.45(0.89-2.35) | 0.14 | 1.54(0.76-3.10) | 0.23 | 1.73(0.73-4.13) | 0.21 |
|  |  | rs3759125 | 1.37(0.86-2.18) | 0.18 | 1.20(0.60-2.37) | 0.61 | 1.91(0.83-4.40) | 0.13 |
|  |  | rs461872 | 0.99(0.44-2.20) | 0.97 | 1.03(0.52-2.00) | 0.94 | 0.96(0.20-4.65) | 0.96 |
|  |  | rs7305534 | 1.04(0.65-1.65) | 0.88 | 1.46(0.71-3.03) | 0.30 | 0.77(0.35-1.68) | 0.51 |
|  |  | rs296766 | 1.50(0.42-5.40) | 0.54 | 1.25(0.61-2.56) | 0.54 | 2.13(0.17-27.40) | 0.56 |
|  |  | rs3759126 | 1.21(0.70-2.08) | 0.49 | 1.18(0.59-2.33) | 0.64 | 1.37(0.50-3.74) | 0.54 |
|  | AQP9 | rs2077737 | 0.87(0.53-1.42) | 0.57 | 0.96(0.50-1.83) | 0.90 | 0.74(0.29-1.88) | 0.52 |
|  |  | rs9920375 | 0.99(0.62-1.58) | 0.97 | 0.90(0.45-1.81) | 0.78 | 1.06(0.46-2.44) | 0.90 |
|  |  | rs1554203 | 0.98(0.78-5.04) | 0.15 | 1.05(0.50-2.18) | 0.90 | 1.07(0.64-26.06) | 0.14 |
|  |  | rs1867380 | 0.60(0.18-2.07) | 0.42 | 1.33(0.66-2.71) | 0.43 | 0.33(0.028-3.76) | 0.37 |
|  |  | rs8023369 | 1.00(0.57-1.74) | 1.00 | 0.82(0.40-1.68) | 0.59 | 1.17(0.43-3.16) | 0.76 |
|  | TMEM205 | rs172731 | 0.54(0.22-1.28) | 0.16 | 0.92(0.48-1.80) | 0.82 | 0.28(0.049-1.57) | 0.15 |
|  |  | rs7251786 | 0.36(0.12-1.08) | 0.068 | 1.60(0.83-3.06) | 0.16 | 0.092(0.010-0.83) | 0.033 |
| No-smoking | OCT2 | rs3823036 | 1.13(0.56-2.27) | 0.73 | 1.28(0.60-2.73) | 0.53 | 1.16(0.30-4.43) | 0.83 |
|  |  | rs2444933 | 2.11(0.84-5.27) | 0.11 | 1.33(0.61-2.88) | 0.47 | 4.21(0.70-25.47) | 0.12 |
|  |  | rs1883306 | 1.02(0.54-1.91) | 0.96 | 0.95(0.42-2.13) | 0.89 | 1.07(0.33-3.51) | 0.91 |
|  | LRP | rs7204252 | 1.26(0.30-5.25) | 0.75 | 1.16(0.40-3.35) | 0.79 | 1.57(0.091-27.25) | 0.76 |
|  |  | rs4788186 | 0.97(0.48-1.99) | 0.94 | 0.99(0.46-2.14) | 0.97 | 0.94(0.23-3.79) | 0.94 |
|  |  | rs1057451 | NA | NA | 2.35(0.92-5.99) | 0.074 | NA | NA |
|  | AQP2 | rs1087598 | 1.10(0.57-2.13) | 0.77 | 1.54(0.67-3.55) | 0.31 | 0.89(0.27-2.95) | 0.85 |
|  |  | rs3759125 | 1.11(0.60-2.04) | 0.74 | 1.20(0.55-2.63) | 0.65 | 1.12(0.36-3.44) | 0.85 |
|  |  | rs461872 | 1.72(0.69-4.26) | 0.24 | 1.54(0.70-3.41) | 0.28 | 2.55(0.43-15.17) | 0.31 |
|  |  | rs7305534 | 0.94(0.55-1.62) | 0.83 | 0.85(0.38-1.91) | 0.70 | 0.98(0.37-2.60) | 0.96 |
|  |  | rs296766 | NA | NA | 0.50(0.18-1.42) | 0.20 | NA | NA |
|  |  | rs3759126 | 1.14(0.65-2.01) | 0.65 | 0.93(0.40-2.13) | 0.85 | 1.47(0.54-4.03) | 0.45 |
|  | AQP9 | rs2077737 | 1.02(0.56-1.86) | 0.94 | 1.18(0.55-2.54) | 0.67 | 1.64(0.57-4.72) | 0.36 |
|  |  | rs9920375 | 0.72(0.40-1.30) | 0.27 | 0.70(0.32-1.54) | 0.37 | 0.58(0.19-1.77) | 0.34 |
|  |  | rs1554203 | NA | NA | 0.88(0.37-2.07) | 0.76 | NA | NA |
|  |  | rs1867380 | NA | NA | 0.43(0.18-1.02) | 0.055 | NA | NA |
|  |  | rs8023369 | 1.53(0.84-2.78) | 0.17 | 1.92(0.81-4.52) | 0.14 | 1.64(0.57-4.72) | 0.36 |
|  | TMEM205 | rs172731 | 0.73(0.30-1.75) | 0.48 | 0.77(0.34-1.72) | 0.52 | 0.56(0.099-3.18) | 0.51 |
|  |  | rs7251786 | 0.83(0.38-1.83) | 0.65 | 0.95(0.43-2.10) | 0.90 | 0.69(0.15-3.22) | 0.64 |
| Smoking | OCT2 | rs3823036 | 0.71(0.43-1.19) | 0.20 | 0.76(0.43-1.34) | 0.35 | 0.56(0.21-1.49) | 0.24 |
|  |  | rs2444933 | 0.56(0.30-1.03) | 0.061 | 0.67(0.38-1.19) | 0.17 | 0.35(0.11-1.15) | 0.084 |
|  |  | rs1883306 | 0.99(0.59-1.65) | 0.96 | 0.94(0.52-1.71) | 0.84 | 1.01(0.37-2.70) | 0.99 |
|  | LRP | rs7204252 | 1.65(0.47-5.79) | 0.43 | 0.97(0.44-2.17) | 0.95 | 2.77(0.22-34.03) | 0.43 |
|  |  | rs4788186 | 1.11(0.63-1.94) | 0.73 | 0.83(0.47-1.47) | 0.51 | 1.38(0.46-4.11) | 0.57 |
|  |  | rs1057451 | NA | NA | 0.76(0.35-1.66) | 0.49 | NA | NA |
|  | AQP2 | rs1087598 | 1.42(0.94-2.14) | 0.098 | 1.40(0.77-2.56) | 0.27 | 1.80(0.86-3.76) | 0.12 |
|  |  | rs3759125 | 1.31(0.87-1.96) | 0.19 | 1.14(0.62-2.07) | 0.68 | 1.77(0.86-3.65) | 0.12 |
|  |  | rs461872 | 1.07(0.56-2.04) | 0.84 | 0.79(0.44-1.43) | 0.44 | 1.28(0.36-4.55) | 0.70 |
|  |  | rs7305534 | 1.23(0.82-1.84) | 0.32 | 1.62(0.85-3.12) | 0.15 | 1.07(0.55-2.07) | 0.85 |
|  |  | rs296766 | 1.26(0.57-2.79) | 0.57 | 1.78(0.96-3.30) | 0.066 | 1.36(0.28-6.62) | 0.71 |
|  |  | rs3759126 | 0.98(0.59-1.61) | 0.92 | 0.98(0.54-1.77) | 0.95 | 0.96(0.38-2.42) | 0.93 |
|  | AQP9 | rs2077737 | 0.80(0.50-1.26) | 0.33 | 0.77(0.44-1.37) | 0.38 | 0.70(0.29-1.68) | 0.43 |
|  |  | rs9920375 | 1.02(0.67-1.55) | 0.94 | 0.88(0.47-1.62) | 0.67 | 1.16(0.55-2.44) | 0.69 |
|  |  | rs1554203 | 1.68(0.71-3.97) | 0.24 | 0.96(0.51-1.78) | 0.89 | 2.95(0.53-16.37) | 0.22 |
|  |  | rs1867380 | 1.25(0.49-3.21) | 0.64 | 1.29(0.70-2.36) | 0.41 | 1.46(0.23-9.51) | 0.69 |
|  |  | rs8023369 | 0.80(0.51-1.27) | 0.35 | 0.62(0.34-1.13) | 0.12 | 0.86(0.37-1.99) | 0.72 |
|  | TMEM205 | rs172731 | 0.78(0.35-1.74) | 0.54 | 0.93(0.52-1.66) | 0.80 | 0.62(0.13-3.03) | 0.56 |
|  |  | rs7251786 | 0.86(0.42-1.75) | 0.68 | 1.14(0.64-2.01) | 0.66 | 0.69(0.17-2.79) | 0.60 |
| Female | OCT2 | rs3823036 | 1.71(0.57-5.19) | 0.34 | 1.02(0.32-3.20) | 0.97 | 0.60(0.19-1.89) | 0.38 |
|  |  | rs2444933 | 2.08(0.53-8.14) | 0.29 | 1.12(0.34-3.64) | 0.85 | 0.60(0.18-1.92) | 0.39 |
|  |  | rs1883306 | 1.04(0.29-3.71) | 0.95 | 1.81(0.48-6.85) | 0.38 | 0.43(0.13-1.42) | 0.17 |
|  | LRP | rs7204252 | NA | NA | 3.86(0.86-17.40) | 0.079 | NA | NA |
|  |  | rs4788186 | NA | NA | 1.88(0.58-6.10) | 0.29 | 0.51(0.15-1.67) | 0.26 |
|  |  | rs1057451 | NA | NA | 2.52(0.60-10.49) | 0.20 | NA | NA |
|  | AQP2 | rs1087598 | 0.84(0.25-2.80) | 0.78 | 1.64(0.48-5.65) | 0.43 | 0.58(0.18-1.81) | 0.34 |
|  |  | rs3759125 | 1.03(0.31-3.43) | 0.96 | 1.13(0.35-3.63) | 0.83 | 0.98(0.099-9.77) | 0.99 |
|  |  | rs461872 | 0.63(0.10-3.89) | 0.62 | 1.18(0.36-3.81) | 0.78 | 0.36(0.0095-13.63) | 0.58 |
|  |  | rs7305534 | 1.00(0.40-2.52) | 0.99 | 2.58(0.70-9.48) | 0.15 | 0.47(0.092-2.41) | 0.37 |
|  |  | rs296766 | NA | NA | 0.83(0.17-3.99) | 0.82 | NA | NA |
|  |  | rs3759126 | 0.54(0.15-1.89) | 0.33 | 1.21(0.33-3.81) | 0.78 | 0.22(0.022-2.27) | 0.21 |
|  | AQP9 | rs2077737 | 1.03(0.44-2.38) | 0.95 | 1.16(0.37-3.60) | 0.80 | 0.97(0.20-4.80) | 0.97 |
|  |  | rs9920375 | 0.69(0.30-1.58) | 0.38 | 0.50(0.16-1.60) | 0.25 | 0.63(0.13-3.05) | 0.57 |
|  |  | rs1554203 | NA | NA | 0.82(0.18-3.66) | 0.79 | NA | NA |
|  |  | rs1867380 | NA | NA | 0.34(0.091-1.29) | 0.11 | NA | NA |
|  |  | rs8023369 | 1.83(0.73-4.54) | 0.19 | 1.79(0.52-6.12) | 0.36 | 2.64(0.51-13.66) | 0.25 |
|  | TMEM205 | rs172731 | 1.81(0.40-8.07) | 0.44 | 0.33(0.084-1.30) | 0.11 | 4.05(0.20-80.23) | 0.36 |
|  |  | rs7251786 | 2.93(0.76-11.25) | 0.12 | 0.38(0.10-1.40) | 0.14 | 11.88(0.82-171.80) | 0.069 |
| Male | OCT2 | rs3823036 | 0.77(0.50-1.20) | 0.25 | 0.85(0.52-1.39) | 0.51 | 0.62(0.27-1.43) | 0.26 |
|  |  | rs2444933 | 0.76(0.46-1.24) | 0.27 | 0.79(0.48-1.30) | 0.35 | 0.61(0.23-1.62) | 0.32 |
|  |  | rs1883306 | 1.06(0.69-1.62) | 0.78 | 0.92(0.55-1.55) | 0.77 | 1.20(0.53-2.69) | 0.66 |
|  | LRP | rs7204252 | 1.49(0.59-3.82) | 0.40 | 0.76(0.37-1.54) | 0.44 | 2.34(0.36-15.18) | 0.37 |
|  |  | rs4788186 | 0.88(0.55-1.39) | 0.57 | 0.70(0.42-1.17) | 0.17 | 0.90(0.37-2.18) | 0.81 |
|  |  | rs1057451 | NA | NA | 0.93(0.48-1.80) | 0.82 | NA | NA |
|  | AQP2 | rs1087598 | 1.37(0.95-1.97) | 0.089 | 1.39(0.82-2.36) | 0.22 | 1.66(0.87-3.17) | 0.12 |
|  |  | rs3759125 | 1.28(0.90-1.82) | 0.17 | 1.18(0.70-1.99) | 0.54 | 1.63(0.87-3.06) | 0.13 |
|  |  | rs461872 | 1.32(0.77-2.25) | 0.32 | 0.97(0.58-1.62) | 0.91 | 1.82(0.63-5.23) | 0.27 |
|  |  | rs7305534 | 1.08(0.76-1.53) | 0.68 | 1.11(0.64-1.93) | 0.72 | 1.10(0.61-1.99) | 0.75 |
|  |  | rs296766 | 1.17(0.53-2.56) | 0.70 | 1.34(0.78-2.30) | 0.29 | 1.26(0.26-6.02) | 0.77 |
|  |  | rs3759126 | 1.16(0.77-1.75) | 0.49 | 0.93(0.55-1.56) | 0.78 | 1.47(0.69-3.15) | 0.32 |
|  | AQP9 | rs2077737 | 0.82(0.55-1.24) | 0.35 | 0.85(0.52-1.41) | 0.53 | 0.71(0.33-1.54) | 0.39 |
|  |  | rs9920375 | 0.95(0.66-1.38) | 0.80 | 0.92(0.54-1.57) | 0.76 | 0.95(0.49-1.85) | 0.89 |
|  |  | rs1554203 | 1.38(0.65-2.90) | 0.40 | 1.01(0.59-1.73) | 0.97 | 1.93(0.44-8.48) | 0.39 |
|  |  | rs1867380 | 1.09(0.47-2.53) | 0.84 | 1.11(0.65-1.89) | 0.71 | 1.15(0.21-6.17) | 0.87 |
|  |  | rs8023369 | 0.89(0.59-1.32) | 0.56 | 0.77(0.45-1.31) | 0.34 | 0.93(0.45-1.92) | 0.84 |
|  | TMEM205 | rs172731 | 0.66(0.35-1.23) | 0.19 | 1.00(0.60-1.66) | 0.99 | 0.42(0.12-1.44) | 0.17 |
|  |  | rs7251786 | 0.72(0.41-1.30) | 0.28 | 1.23(0.75-2.02) | 0.42 | 0.46(0.15-1.44) | 0.18 |
